# Nature Relatedness and Environmental Concern of Young People in Ecuador and Germany

**DOI:** 10.3389/fpsyg.2019.00453

**Published:** 2019-03-07

**Authors:** Maximilian Dornhoff, Jan-Niklas Sothmann, Florian Fiebelkorn, Susanne Menzel

**Affiliations:** Faculty of Biology/Chemistry, Osnabrück University, Osnabrück, Germany

**Keywords:** biodiversity, students, values, cross-cultural, environmental concern, gender, sustainable development, self-transcendence

## Abstract

Today’s societies are confronted by a daily biodiversity loss, which will increase in the face of climate change and environmental pollution. Biodiversity loss is a particularly severe problem in so-called biodiversity hotspots. Ecuador is an example of a country that hosts two different biodiversity hotspots. Human behavior – in developing as well as in industrial countries such as Germany – must be considered as one of the most important direct and indirect drivers of this global trend and thus plays a crucial role in environmentalism and biodiversity conservation. Nature relatedness and environmental concern have been identified as important environmental psychological factors related to people’s pro-environmental behavior. However, the human–nature relationship depends on a variety of other factors, such as values, gender, nationality, qualities of environmental concern and time spent in nature. This study compared young people from Ecuador and Germany with regard to their nature relatedness and environmental concern. Furthermore, the role of the aforementioned factors was investigated. In total, we surveyed 2,173 high school students from Germany (*M*_age_ = 14.56 years, *SD* = 1.45; female: 55.1%) and 451 high school students from Ecuador (*M*_age_ = 14.63 years, *SD* = 1.77; female: 55.3%). We found that young Ecuadorians were more related to nature than young people from Germany. Additionally, we found country-specific differences in the structure of environmental concern and in the role of gender in the explanation of biospheric environmental concern and nature relatedness. In both samples, the self-transcendence value cluster was a significant positive predictor for biospheric environmental concern and nature relatedness. Time spent in nature was a significant positive predictor for nature relatedness in both samples. The results are an empirical basis for the assumption of culture-specific differences in human–nature relationships.

## Introduction

The rate of biodiversity loss, among other environmental problems, such as climate change and biogeochemical cycles, has already exceeded its safe planetary boundary (Rockström et al., 2009; Steffen et al., 2015). Biodiversity loss not only affects the functioning of ecosystems (Cardinale et al., 2012) but also the ecosystem services for humanity (Costanza et al., 1997; Millenium Ecosystem Assessment, 2005). Even though the negative consequences of environmental destruction are globally relevant, some ecoregions, especially biodiversity hotspots, are of particular importance in terms of biodiversity conservation. These biodiversity hotspots are characterized by an extraordinary plant and animal endemism as well as high levels of habitat loss (Mittermeier et al., 2011). By definition a hotspot must contain at least 1,500 endemic plant species (0.5% of the worlds’ plant species) and should have lost at least 70% of its primary vegetation (Myers et al., 2000).

Ecuador is extremely rich with respect to biodiversity, as it is covered by two biodiversity hotspots, namely, the Tumbes-Chocó-Magdalena and Tropical Andes Hotspot (Mittermeier et al., 2011). For instance, Ecuador has the highest density of vertebrates species in the world (Myers et al., 2000). It hosts about 7.3% of the vertebrate species described worldwide and 7.6% of the vascular plant species (Ministerio del Ambiente del Ecuador, 2015). However, Ecuador is a so-called developing country in which a large part of the population suffers drastic socio-economic inequalities (Lopez-Cevallos and Chi, 2010). Therefore, socio-economic development is required, which is often considered to be associated with environmental degradation (Panayotou, 2016). For instance, Ecuador is still reporting a decrease in forest area (Food and Agriculture Organization of the United Nations [FAO], 2016) and an increased number of endangered species (Ministerio del Ambiente del Ecuador, 2015). The International Union for Conservation of Nature and Natural Resources (International Union for Conservation of Nature [IUCN], 2017a) lists nine extinct and 518 critically endangered, endangered, or vulnerable animal species, whereas nine plant species are considered to be extinct, and 1,857 plant species are classified as critically endangered, endangered, or vulnerable (International Union for Conservation of Nature [IUCN], 2017b).

However, the Ecuadorian government has developed new approaches for sustainable development with a focus on biodiversity conservation. For instance, Ecuador was the first country to incorporate the rights of nature and the indigenous concept of *Buen Vivir* in its constitution (Asamblea Constituyente de Ecuador, 2008). The basic idea of *Buen Vivir* is the good way of living in harmony with nature and other human beings (Lalander, 2016). However, Ecuadorian state policies are characterized by economic interests that are hindering the effective implementation of new biodiversity conservation measures (Lalander, 2016). Nevertheless, the debate about *Buen Vivir* and the rights of nature has contributed to a growing socio-cultural awareness regarding environmental issues (Rieckmann et al., 2011; Lalander, 2016). In addition, in industrialized countries such as Germany, efforts are being made to adapt some aspects of the basic concept of *Buen Vivir* (Acosta, 2015).

In a worldwide comparison, Germany, one of the most industrialized countries in the world, is counted as an area with relatively low biodiversity, on the basis of geological history development and geographic location. For instance, it hosts only 1.2% of the vertebral species described worldwide and 1.4% of the vascular plant species (Federal Ministry for the Environment, Nature Conservation and Nuclear Safety [BMU], 2014). Additionally, the Federal Ministry for the Environment Nature, Conservation and Nuclear Safety observed a statistically significant deterioration of species diversity (Federal Ministry for the Environment, Nature Conservation and Nuclear Safety [BMU], 2014). In Germany, four animal species are considered to be extinct and 101 critically endangered, endangered, or vulnerable (International Union for Conservation of Nature [IUCN], 2017a). With regard to plant species richness, 36 species are considered to be critically endangered, endangered, or vulnerable (International Union for Conservation of Nature [IUCN], 2017b). Thus, Germany and Ecuador are both affected by a progressive loss of species.

To reduce biodiversity loss, both countries have drafted a national biodiversity strategy with ambitious goals regarding biodiversity conservation (Federal Ministry for the Environment, Nature Conservation and Nuclear Safety [BMU], 2007; Ministerio del Ambiente del Ecuador, 2016). Furthermore, Germany cooperates internationally to support biodiversity on a global scale (Federal Ministry for the Environment, Nature Conservation and Nuclear Safety [BMU] and Federal Ministry for Economic Cooperation and Development [BMZ], 2016). Both countries have acknowledged human behavior as core challenge in all efforts to protect biodiversity. Thus, in addition to political efforts to conserve biodiversity, human behavior, and thus, people’s attitudes and values are becoming increasingly significant worldwide in preserving biodiversity (Federal Ministry for the Environment, Nature Conservation and Nuclear Safety [BMU], 2007; Nisbet and Zelenski, 2013). Besides the aforementioned personality traits that may be related to environmental behavior, it seems that people – and especially young people – have lost their inner connection with nature due to modern societal development that hinders a human–nature interaction (Louv, 2008; Brämer et al., 2016; Soga and Gaston, 2016). A disturbed human–nature relationship, however, has been considered one of the main reasons for people’s harmful behavior toward the environment and for decreasing environmental concern (Nisbet and Zelenski, 2013). Given that young people are approaching the stage of taking responsibility for their own lives, including a critical reflection of their own environmental behavior, these results seem particularly alarming. Moreover, young people are in an important period of value formation (Wray-Lake et al., 2010). As they are still students, appropriate educational programs can help to promote the formation of important values fostering pro-environmental behavior (von Braun, 2017). Regarding the impact of environmental education, Otto and Pensini (2017) showed that the frequency of children’s visits to nature-based environmental education institutions is positively related to pro-environmental behavior, mediated by an increased environmental knowledge and nature relatedness. Furthermore, they found nature relatedness to explain a high percentage and environmental knowledge a low percentage of the variance in pro-environmental behavior (Otto and Pensini, 2017).

Nature relatedness can be understood as a perceived cognitive, affective, and experiential connection to the natural world that is regarded to be “trait-like,” because it is relatively stable over time and across situations but not completely fixed (Schultz, 2002; Mayer and Frantz, 2004; Nisbet et al., 2009; Brügger et al., 2011; Nisbet and Zelenski, 2013). The cognitive component of nature relatedness can be considered as the extent to which people include nature within their cognitive representation of self, which in turn is regarded as the fundamental aspect of human–nature relations by some authors (Schultz, 2002). Other authors place the affective connection, the sense of feeling connected, at the center of the human–nature relationship (Mayer and Frantz, 2004). The experiential connection is often neglected but is an important aspect in some concepts of nature relatedness (Nisbet et al., 2009). It represents an individual’s physical familiarity with the natural world and the level of perceived comfort with being in nature. Since we refer to the nature relatedness construct of Nisbet et al. (2009) all three aspects of individual connection with the natural world are regarded as equally important.

Nature relatedness can be explained theoretically by the biophilia hypothesis (Wilson, 1984) that assumes an innate tendency of humans to approach and contact other living and natural things. The biophilia hypothesis postulates that it is inherent in human beings to learn from and value the natural environment (Kellert and Wilson, 1993).

Additionally, studies have shown that having frequent nature contact promotes nature relatedness and may lead to increased environmental concern (Mayer et al., 2009; Nisbet and Zelenski, 2013) and that nature-related people spend more time outdoors in a natural environment (Nisbet et al., 2009; Raymond et al., 2010). Moreover, Kals et al. (1999) found the frequency of time spent in nature to be a powerful predictor for emotional affinity toward nature.

In addition to nature relatedness, environmental concern plays an important role in explaining environmental behavior. As part of their ‘value basis of environmental concern’ theory, Stern and Dietz (1994) suggest that environmental concern can be based on egoistic, social-altruistic, and biospheric value orientations and on beliefs about the consequences of environmental changes for valued objects. Based on this, Schultz (2001) could show a three factorial structure of environmental concern. These three factors are egoistic concern, altruistic concern, and biospheric concern about the environment, depending on whether individuals care about themselves, other people, or all living things. Thus, he explained that one person’s environmental concern and behavior are not necessarily based on their nature relatedness but may have egoistic or altruistic motives (Schultz, 2002). Yet a positive relation to pro-environmental behavior could only be demonstrated for biospheric concern about the environment (Schultz, 2001). Stern et al. (1993) showed that women have stronger beliefs about the harmful consequences of bad environmental conditions for self, others, and the biosphere and that pro-environmental behavior is predicted by these beliefs. These gender differences are attributed to socialization processes (Gilligan, 1982; Beutel and Marini, 1995; Baron-Cohen and Wheelwright, 2004; Jolliffe and Farrington, 2006) that are regarded as culture-specific (Davidson and Freudenburg, 1996).

Value orientations determine the actions of people, their beliefs, and attitudes in general as well as toward nature (Schwartz, 1994; Schultz et al., 2004). In order to explain value-based environmental behavior in cross-cultural studies, the Schwartz theory of basic human values (Schwartz, 1992, 1994) has proven to be particularly appropriate since certain values could be identified in more than 30 nations. The basic human Schwartz-values of the self-transcendence value cluster have proven to be a powerful predictor for a connection to nature (Sothmann and Menzel, 2017). The self-transcendence value cluster represents prosocial norms oriented toward the welfare of close others in everyday interaction humans (benevolence) and all people and nature including all living beings (universalism; Schwartz, 1992). Furthermore, it correlated positively with biospheric and altruistic concern but negatively with egoistic concern about the environment (Schultz, 2001; Schultz et al., 2005). The basic human values of the self-enhancement value cluster showed a positive relation with egoistic environmental concern but a negative relation with biospheric and altruistic concern (Schultz, 2001; Schultz et al., 2005). It represents values orientated toward success, the demonstration of competence (achievement), social status and prestige (power), pleasure and enjoyment of life (hedonism) (Schwartz, 1992).

Up to now, there has been no comparison of young people from Latin America and those from Europe with regard to their nature relatedness and environmental concern and the factors that predict them. Therefore, the present study aims at providing insight into the relatively unexplored topic of intercultural differences of young people’s human–nature relationship.

### The Present Study

When considering biodiversity and its loss globally, we assume that Ecuadorian young people, who live in a biodiversity hotspot, and German young people, who grow up in one of the most industrialized countries in the world, show differences in their human–nature relationship. Our assumption is reinforced by studies that show that Ecuadorian college students score higher on environmental concern than United States and European college students (Schultz, 2001). Regarding an international comparison of nature relatedness, there is insufficient empirical evidence to develop a literature-based hypothesis. However, due to the higher biodiversity in Ecuador and the fact, that the Ecuadorian people triggered current political debate on the rights of nature, we assume that Ecuadorian people in general as well as young people are higher in their nature relatedness than German young people. Additionally, we are interested in the factors that are related to nature relatedness and environmental concern. Based on the aforementioned studies, we expect self-transcendence, time spent in nature, and having a female gender to positively predict nature relatedness and biospheric environmental concern in both samples. A cross-cultural investigation into the relations between young people’s nature relatedness, environmental concern, and the basic human values of the self-transcendence value cluster should provide important information that could be used to design adequate environmental education and outreach projects in both countries.

Thus, the present study aimed at comparing Ecuadorian and German young people’s nature relatedness and environmental concern and at elucidating the factors that are related to them.

Three research questions and subsequent hypotheses were addressed:

Q1: How do Ecuadorian and German young people differ in their nature relatedness and environmental concern?

H1: Ecuadorian young people show higher nature relatedness and environmental concern.

Q2: How do young people’s gender and nationality, their basic human values, and time spent in nature affect their nature relatedness and environmental concern?

H2: Self-transcendence, time spent in nature, and having a female gender positively predict nature relatedness and biospheric environmental concern.

H3: Self-transcendence positively predicts altruistic concern and negatively egoistic concern.

H4: Self-enhancement positively predicts egoistic environmental concern.

## Materials and Methods

### Participants and Procedure

The sample was divided into two subsamples. The first sample consisted of 2,173 anonymously surveyed high school students from ten Northwest Germany secondary schools in and around the cities of Osnabrück and Hanover (*M*_age_ = 14.56 years, *SD* = 1.45; female: 55.1%). Five schools were located on the outskirts of the city, close to forest areas or agricultural land. In contrast, five schools were located in the center of the city, without direct access to forest areas or agricultural land. The second sample consisted of 451 anonymously surveyed high school students from four private secondary schools located in Southern Ecuador in and around the city of Cuenca (*M*_age_ = 14.63 years, *SD* = 1.77; female: 55.3%). While one school was located on the outskirts of the city, close to forest areas and agricultural land, three schools were located in the center of the city, far from forest areas or agricultural land.

The variables addressed in this article were embedded in a paper-and-pencil questionnaire. The survey contained 66 items and was conducted during regular school hours. The students had the length of one school lesson (45 min) to complete the questionnaire. The time limit was not exceeded in any case. Some students finished the questionnaire 15 min before the end of the time limit. Without measuring the average time precisely, we can conclude from our classroom observations that the Ecuadorian students needed more time to complete the questionnaire than the German students. The differences between the two samples in the time taken to complete the questionnaire can be explained by the differences in reading literacy between Latin American and German students. The assumption that the German sample achieved a higher level of reading skills than the Ecuadorian sample is based on large-scale assessments in education (OECD, 2016). Of course, this is only one possible explanation. It may be the case that Ecuadorian students paid closer attention to the questions than the German students did.

Anonymity was guaranteed, and participation was voluntary. Ethical approval for the study was obtained in July 2016 from the responsible State Board of Education in Germany - Niedersächsische Landesschulbehörde (NLSchB), which is the body responsible for providing ethics approvals for studies carried out in schools. The participating German schools were informed about the research conducted and provided their written consent. All participants had the chance to decline to participate and to withdraw from the research at any time. Since we surveyed Ecuadorian students from private schools, we asked the respective headmasters, in advance, for permission to carry out the questionnaire study. In all schools, the questionnaire was reviewed by the school psychologist, who did not raise any objections to the study. Permission from the headmasters was granted for all schools.

In both countries, the parents of the students were informed about the study by a letter, in which the voluntary participation and anonymity of the study was pointed out. The possibility to contact us was given by the attached contact data. According to the APA’s Ethical Principles of Psychologists and Code of Conduct (American Psychological Association, 2016) psychologists may dispense with informed consent where research would not reasonably be assumed to create distress or harm. As our investigation was conducted by an anonymous questionnaire in an educational setting and in the presence of the respective teacher, an informed consent was not necessary (American Psychological Association, 2016). Moreover, the responsible State Board of Education in Germany only requires written consent in the case of surveys involving the processing of personal data. However, this was not the case in the present study. Furthermore, since the students were not asked about their parents or family circumstances, racial and ethnic origin, political opinions, religious beliefs, health, or sex life, no informed consent of the legal guardian is required (Niedersächsische Landesschulbehörde, 2015). The consent procedures followed were also approved by the State Board of Education in Germany – Niedersächsische Landesschulbehörde (NLSchB).

### Materials

We measured the amount of time spent in nature as a basic socio-demographic sample characteristic and used established psychometric scales to assess altruistic, egoistic, and biospheric concern about the environment as well as nature relatedness and value orientations.

*Time spent in nature* was measured by one item asking how much time the participants generally spend in nature. They answered on a 5-point Likert scale ranging from 1 (*very little*) to 5 (*very much*). We deliberately refrained from providing a definition of nature and an exact indication of time, as several studies have already shown that humans can have very different concepts of nature. For example, an artificial park can be viewed as nature for a person from the city, whereas a cultural landscape with farmlands can represent nature for a person from the countryside (Thompson et al., 1990; Kleinhückelkotten and Neitzke, 2010). Thus, we preferred to assess students’ subjective perception of nature. In addition, some people may have easier access to nature than others, which might influence their perception of the time they spent in nature. For instance, for a person who lives and works in an urban environment, 20 min per day in a park may be a lot of valuable time in nature, whereas for a person from the countryside, 20 min in a forest may not be considered very much time. We intended to address these potential differences between the subjective conception of nature and time by asking in this way. Nevertheless, this single item is a relatively soft indicator of time spent in nature, which should be taken into account when interpreting the results.

The Environmental Concern Scale developed by Schultz (2001) is an established instrument for measuring concern about the environmental problems rooted in human behavior. Following the original scale as suggested by Schultz (2001), 12 items were used to ask participants whether their environmental concern is caused by egoistic, altruistic, or biospheric motives. Participants rated each of the items from 1 (*not important*) to 5 (*important*) on a 5-point Likert scale. The scale starts with the following statement:

‘People around the world are generally concerned about environmental problems because of the consequences that result from harming nature. However, people differ in the consequences that concern them the most. How important are the consequences of environmental problems for…?’

Each dimension of environmental concern was measured by four items: egoistic concern by (1) me, (2) my lifestyle, (3) my health, and (4) my future; altruistic concern by (1) people in my community, (2) all people, (3) children, and (4) future generations; and biospheric concern by (1) plants, (2) marine life, (3) birds, and (4) mammals. We created the German version of the scale by translation and back-translation. For the Ecuadorian sample, we mainly used the Spanish version by Schultz (2001). In both the German and Spanish versions, we replaced the original biospheric concern item (4) *animals* with *mammals* to illustrate the difference to (3) *birds*. After consultation with native speakers familiar in local dialects, we replaced the original Spanish altruistic concern item (4) *mis paisanos* by *mis compatriotas*, because the latter is more commonly used in the region. Exploratory factor analyses showed that the three environmental concern dimensions loaded on their theoretically separate factors with high reliabilities for both samples (Table 1).

**Table 1:** Reliabilities, results of the Kolmogorov–Smirnov test, and sources of the scales used in the current study.

Scale	Germany			Ecuador			Items
			
	α	*n*	K-S	α	*n*	K-S	
ST^1^	0.72	2,048	0.09^∗∗∗^	0.72	432	0.13^∗∗∗^	8
SE^1^	0.77	2,065	0.06^∗∗∗^	0.72	432	0.06^∗∗∗^	7
NR-6^2^	0.80	2,001	0.06^∗∗∗^	0.83	426	0.10^∗∗∗^	6
EC^3^	0.86	2,064	0.07^∗∗∗^	0.85	371	0.14^∗∗∗^	12
Egoistic EC	0.77	2,107	0.10^∗∗∗^	0.79	425	0.20^∗∗∗^	4
Altruistic EC	0.78	2,100	0.13^∗∗∗^	0.72	388	0.17^∗∗∗^	4
Biospheric EC	0.92	2,115	0.14^∗∗∗^	0.91	434	0.26^∗∗∗^	4


*ST, self-transcendence; SE, self-enhancement; EC, environmental concern; ^*1*^Source: García Castro (2014) for the Spanish version, Schmidt et al. (2007) for the German version. ^*2*^Source: Nisbet and Zelenski (2013) for the English version; ^*3*^Source: Schultz (2001) for the Spanish and English version, ^∗∗∗^*p* ≤ 0.001.*

The self-transcendence and the self-enhancement values were measured by eight and nine items from the Portrait Values Questionnaire (Schmidt et al., 2007), which is composed of verbal portraits defining a person’s goals, expectations, or desires that implicitly indicate the importance of a value. Respondents were asked to rate the similarity of the described person to themselves on a 5-point Likert scale ranging from 1 (*not like me at all*) to 5 (*very much like me*). For the Ecuadorian sample, we used an approved Spanish version of the scale (García Castro, 2014). A cross-cultural construct validity for the Portrait Values Questionnaire could be confirmed in various studies (Schwartz and Sagiv, 1995; Spini, 2003).

There are numerous suitable measures of subjective connectedness with the natural environment. For instance, the Disposition to Connect with Nature Scale (Brügger et al., 2011) is an intellectually simple instrument consisting of 40 items that relies only on simple self-reflection and is therefore well suited to assess the nature relatedness of school students (Brügger and Otto, 2017). In order to avoid respondent fatigue, we decided to measure nature relatedness via the much shorter 6-item version of the Nature Relatedness Scale (NR-6; Nisbet and Zelenski, 2013). Participants were asked to what extent they agreed with statements like ‘I feel very connected to all living things and the earth’ on a 5-point Likert scale ranging from 1 (*I disagree*) to 5 (*I agree*). The German as well as the Spanish version of the scale were created by translation and back-translation and checked by native speakers familiar with local dialects and the scale.

Even though the scales used in this study were originally designed for adults, the Portrait Values Questionnaire has already been validated with young people. For instance, Menzel and Bögeholz (2010) validated the Portrait Values Questionnaire by surveying an international sample of 15- to 19-year-old Chilean and German school students. It is regarded as a relatively intellectually less demanding instrument for measuring human values (Schmidt et al., 2007). There are no known studies using the environmental concern scale and the NR-6 on a comparably young sample. In addition, the current study found good reliability for both scales.

### Analyses

First, we conducted exploratory factor analyses in order to empirically test the scales used for the two samples on dimensionality. According to the theoretical basis, the tested were regarded as interdependent, which is why we performed oblimin rotation. Additionally, we conducted a confirmatory factor analysis in order to verify the factor structure of the environmental concern scale. We then checked our scales for normality with a Kolmogorov–Smirnov test and computed reliability with Cronbach’s alpha.

With regard to the Portrait Values Questionnaire, we decided to exclude two items of the hedonism value type, which were to be assigned theoretically to the value dimension of self-enhancement, because in the German sample, the items SEHE1 and SEHE3 loaded on the second (self-transcendence) factor. In the Ecuadorian sample, only SEHE3 did so (see Table 2). An explanation for this can be found in the dynamic structure of value types presented by Schwartz (1992). He points out that despite the focus of hedonism on self, it is not characterized by the same competitive motivation that is expressed by achievement and power values. Moreover, hedonism is apparently characterized by the motivation for arousal and challenge, which is not represented in achievement and power since they show a frequent proximity to the conservation value dimension (Schwartz, 1992).

**Table 2:** Factor loadings based on an exploratory factor analysis with oblimin rotation for 17 items from the Portrait Values Questionnaire (PVQ) (*N*_Germany_ = 1,965; *N*_Ecuador_ = 411).

Items for the collected value types	Germany		Ecuador	
		
	SE	ST	SE	ST
SEPO1: It is important to him/her^1^ to be rich. He/She wants to have a lot of money and expensive things.	**0**.**57**	-0.25	**0**.**52**	-0.12
SEPO2: It is important to him/her to be in charge and tell others what to do. He/She wants people to do what he/she says.	**0**.**68**	-0.26	**0**.**60**	-0.24
SEPO3: He/She always wants to be the one who makes the decisions. He/She likes to be the leader.	**0**.**71**	-0.19	**0**.**67**	0.03
SEAC1: It is very important to him/her to show his/her abilities. He/She wants people to admire what he/she does.	**0**.**64**	0.04	**0**.**57**	0.13
SEAC2: Being very successful is important to him/her. He/She likes to impress other people.	**0**.**69**	0.00	**0**.**67**	0.08
SEAC3: Getting ahead in life is important to him/her. He/She strives to do better than others.	**0**.**70**	-0.07	**0**.**60**	0.07
SEHE1: He/She seeks every chance he/she can to have fun. It is important to him/her to do things that give him/her pleasure^∗^.	0.24	**0**.**52**	**0**.**40**	0.35
SEHE2: Enjoying life’s pleasures is important to him/her. He/She likes to ‘spoil’ himself/herself.	**0**.**47**	0.30	**0**.**57**	0.38
SEHE3: He/She really wants to enjoy life. Having a good time is very important to him/her^∗^.	0.31	**0**.**47**	0.29	**0**.**49**
STUN1: He/She thinks it is important that every person in the world be treated equally. He/She believes everyone should have equal opportunities in life.	-0.15	**0**.**54**	-0.01	**0**.**54**
STUN2: It is important to him/her to listen to people who are different from him/her. Even when he/she disagrees with them, he/she still wants to understand them.	-0.12	**0**.**55**	-0.08	**0**.**46**
STUN3: He/She strongly believes that people should care for nature. Looking after the environment is important to him/her.	-0.06	**0**.**46**	0.13	**0**.**59**
STUN4: It is important to him/her to adapt to nature and to fit into it. He/She believes that people should not change nature.	-0.05	**0**.**40**	0.08	**0**.**50**
STBE1: It’s very important to him/her to help the people around him/her. He/She wants to care for other people.	-0.13	**0**.**66**	0.03	**0**.**66**
STBE2: It is important to him/her to be loyal to his friends. He/She wants to devote himself to people close to him.	0.03	**0**.**66**	0.07	**0**.**51**
STBE3: It is important to him/her to respond to the needs of others. He/She tries to support those he knows.	-0.05	**0**.**71**	0.08	**0**.**70**
STBE4: Forgiving people who might have wronged him/her is important to him/her. He/She tries to see what is good in them and not to hold a grudge.	-0.20	**0**.**42**	-0.20	**0**.**44**
**Factor correlations between SE and ST**	-0.05		0.08	


*^1^In the German version, we used “the person” instead of “he/she” and “him/her.” Factor loadings ≥0.4 are printed in bold. Items marked with asterisk (^∗^) will not be included in further analyses. SE, self-enhancement; ST, self-transcendence, PO, power; AC, achievement; HE, hedonism; UN, universalism; BE, benevolence.*

Confirmatory factor analysis verified the three-factor structure of environmental concern (see Supplementary Material). All scales showed acceptable, good to very good reliabilities for both samples (Table 1). To answer our research questions, we included a total of 27 items from the aforementioned scales in our analyses.

Although some variables did not follow a normal distribution, we calculated independent group *t*-tests to compare the German and the Ecuadorian samples. However, we interpreted the bootstrap with 95% bias corrected and accelerated confidence intervals as recommended by Field (2017) in the case of non-normal distributed variables. Since it is a cross-cultural study, a response bias cannot be ruled out (Hofstede, 1980; Smith, 2004; Schwartz, 2009), which is why we also carried out standardized mean value comparisons, using the method of group mean centering (Fischer, 2004). For creating scores that controlled for differences in response tendency, we produced group-mean centered egoistic, altruistic, and biospheric environmental concern scale scores by subtracting the group mean of all 12 of the environmental concern items (EC-mean_Germany_ = 4.01; EC-mean_Ecuador_ = 4.42) from each of the three scale scores (see also Schultz et al., 2004). Furthermore, we computed the grand mean of all the items of the value clusters self-transcendence and self-enhancement (we only asked for these two value clusters). Afterward, we subtracted the total of all 14 items (PVQ-mean_Germany_ = 3.44; PVQ-mean_Ecuador_ = 3.62) from the scale score of self-transcendence and self-enhancement (see also Schwartz, 2009). The mean-corrected scores are presented in the lower part of Table 3. The effect sizes of group differences were calculated by Cohen’s *d*, using the two means (raw mean scores and centered mean scores), standard deviations, and the sample sizes of both groups (Hedges and Olkin, 1985).

**Table 3:** Comparison between the mean scores of the German and Ecuadorian samples.

Variables	Germany			Ecuador			*t*-test	95% BCaCI	Effect size *d*
					
	*M*	*SE*	*SD*	*M*	*SE*	*SD*			
Nature relatedness	2.66	0.02	0.78	3.69	0.04	0.83	-24.54^***^	[-1.12, -0.95]	1.32
Time spent in nature	2.91	0.02	0.88	2.82	0.04	0.88	1.95^*^	[0,00, 1.18]	0.10
Egoistic EC	-0.14	0.02	0.74	0.02	0.03	0.65	-3.53^***^	[-0.20, -0.05]	0.17
Altruistic EC	0.11	0.02	0.73	-0.13	0.04	0.71	6.15^***^	[0.17, 0.32]	0.33
Biospheric EC	0.00	0.02	0.91	0.08	0.04	0.72	-2.01^*^	[-1.61, -0.01]	0.09
ST	0.39	0.01	0.55	0.47	0.03	0.62	-2.55^**^	[-0.15, -0.02]	0.14
SE	-0.44	0.02	0.69	-0.57	0.04	0.74	3.42^**^	[0.05, 0.21]	0.19


*EC, environmental concern; ST, self-transcendence; SE, self-enhancement. Confidence intervals based on 1,000 bootstrap samples, ^∗^*p* ≤ 0.05, ^∗∗^*p* ≤ 0.01, ^∗∗∗^*p* ≤ 0.001.*

In order to answer the second research question, we conducted a robust multiple regression, because some scales followed a non-normal distribution. After that, we compared the resulting *b-*values, the standard errors, and the *t-*statistics with the non-robust versions. The robust estimates revealed basically the same results; hence we report the non-robust versions, as recommended by Field (2017). Since we were interested in the effect of young people’s socio-demographic factors and values on their nature relatedness and environmental concern, we calculated regression analyses for the independent variables nature relatedness as well as egoistic, altruistic, and biospheric environmental concern for both samples.

## Results

**Q1: How do Ecuadorian and German young people differ in their nature relatedness and environmental concern?**

The results of the independent group *t*-tests are reported in Table 3. Since a centering was not possible for nature relatedness and time spent in nature, uncentered scores are reported for these variables. For environmental concern, self-transcendence and self-enhancement, only the centered scores are provided (see Supplementary Material for presentation of uncentered scores).

Regarding nature relatedness, the *t*-test revealed differences with large effect sizes between German and Ecuadorian young people, with Ecuadorians scoring higher than Germans.

The comparison between the centered mean scores showed only altruistic environmental concern as differing significantly between the two groups, with a small effect size. In this case, German young people scored higher than Ecuadorians. Additionally, the centered mean score comparison provided insight into the structure of environmental concern for our two samples. Whereas we found a relative preference for altruistic (*M* = 0.11) over biospheric (*M* = 0.00) and egoistic concern (*M* = -0.14) in the German sample, the Ecuadorian sample was most concerned about the consequences of environmental problems for biospheric reasons (*M* = 0.08), followed by egoistic (*M* = 0.02) and altruistic reasons (*M* = -0.13).

**Q2: How do young people’s gender and nationality, their basic human values, and time spent in nature affect their nature relatedness and environmental concern?**

Multiple regressions were conducted in order to determine how the sample’s gender, their values, and time spent in nature affected their nature relatedness and environmental concern. To investigate the differences between both samples in explaining nature relatedness and environmental concern, we carried out separate multiple regressions for our two groups (Table 4).

**Table 4:** Results of regression analyses predicting nature relatedness as well as egoistic, altruistic, and biospheric environmental concern for the German and the Ecuadorian sample.

	NR		Egoistic EC		Altruistic EC		Biospheric EC	
				
	β	*t*	β	*t*	β	*t*	β	*t*
**German sample**								
ST	0.37	17.91^***^	0.26	11.24^***^	0.42	18.98^***^	0.40	18.17^***^
SE	-0.01	-0.62	0.14	6.33^***^	0.01	0.53	0.01	0.43
Time spent in nature	0.34	16.79^***^	0.08	3.41^***^	0.02	0.97	0.06	2.90^*^
Female	0.12	5.73^***^	-0.02	-0.65	-0.01	-0.49	0.00	0.13
Adj. *R*^2^	0.30^∗∗∗^		0.09^∗∗∗^		0.18^∗∗∗^		0.18^∗∗∗^	
*N*	1,820		1,910		1,904		1,912	
**Ecuadorian sample**								
ST	0.32	7.15^***^	0.17	3.45^**^	0.31	5.94^***^	0.25	5.04^***^
SE	-0.03	-0.58	0.19	3.74^***^	-0.01	-0.18	-0.03	-0.53
Time spent in nature	0.31	6.90^***^	0.08	1.55	-0.02	-0.41	0.14	2.93^**^
Female	-0.11	-2.42^*^	-0.01	-0.15	0.01	0.14	-0.13	-2.56^*^
Adj. *R*^2^	0.24^∗∗∗^		0.07^∗∗∗^		0.08^∗∗∗^		0.11^∗∗∗^	
*N*	390		387		356		395	


*ST, self-transcendence; SE, self-enhancement; NR, nature relatedness; EC, environmental concern,^∗^*p* ≤ 0.05, ^∗∗^*p* ≤ 0.01, ^∗∗∗^*p* ≤ 0.001.*

In both samples, self-transcendence and time spent in nature showed a positive effect on nature relatedness. Whereas female gender in the German sample predicted the nature relatedness positively, the reverse was true in the Ecuadorian sample. Neither in the German sample nor in the Ecuadorian sample did self-enhancement have an effect on nature relatedness. The regression explained 30% of nature relatedness’ variance in the German sample and 24% in the Ecuadorian sample.

Furthermore, multiple regressions accounted for 9% of egoistic concerns’ variance in the German sample and 7% in the Ecuadorian sample. In both samples, self-transcendence and self-enhancement showed a positive effect on egoistic concern. In both samples, only self-transcendence predicted altruistic concern. The regression on altruistic concern explained 18% of its variance in the German sample and 8% in the Ecuadorian sample.

In both samples, self-transcendence and time spent in nature had a positive effect on biospheric concern. While there was no relation between female gender and biospheric concern in the German sample, female gender showed a negative effect on biospheric concern in the Ecuadorian sample. The regression on biospheric concern explained 18% of the variance in the German sample and 11% in the Ecuadorian sample.

## Discussion

**Q1: How do Ecuadorian and German young people differ in their nature relatedness and environmental concern?**

**H1: Ecuadorian young people show higher nature relatedness and environmental concern.**

With our first research question, we intended to compare young people’s nature relatedness and environmental concern between the two samples from Ecuador and Germany.

In a comparison of means across different cultures, a response bias cannot be ruled out, because people from different cultures differ in their response behavior (Smith, 2004) and socially desirable responding influences the self-reported priorities (Schwartz et al., 1997). For this reason, we consider the standardized mean scores (Table 3) to be more meaningful and to better represent the priorities of their values and environmental concern than the non-standardized values. Thus, regarding environmental concern, we decided to report only the comparison of the centered mean scores. The discussion of the differences in nature relatedness refers to the raw scores.

A deeper look into the structure of environmental concern revealed clear patterns in each sample. The prioritization of altruistic concern in the structure of environmental concern, which was the case in the German sample, was frequently found, for example, in nine of eleven adult samples from the United States and different Latin American countries surveyed by Schultz (2001). Only El Salvador and Columbia were most concerned about the consequences of environmental problems for biospheric reasons. However, a German sample was not part of the study mentioned above.

We suspect that living in the biodiversity hotspot Tropical Andes influences Ecuadorian young people’s environmental concern, thus presenting a possible explanation for the Ecuadorian young people’s structure of environmental concern. A biodiversity hotspot is characterized not only by its high species density but also by its high degree of threat. The biodiversity in such a place is therefore particularly worth protecting and people living there could be more aware of nature’s intrinsic value, which could explain the higher biospheric concern of Ecuadorian young people.

Regarding egoistic and altruistic environmental concern, the occurrence and consequences of environmental disasters, which differ extremely in Ecuador and Germany, have to be considered. Ecuadorians live in a biodiversity hotspot and news like the destruction of tropical rainforests for the exploration of oil or the cultivation of crops destined for export to Europe is not uncommon. Many human-made environmental problems have either a direct or indirect consequence on their personal lives, whether through land loss, water pollution, or the loss of traditional food and medicinal plants. For instance, during oil exploitations in the Ecuadorian Amazon by an American multinational energy corporation between 1964 and 1992, millions of gallons of toxic substances were spilled into the Amazon. The contamination covered an area of 1,700 square miles and caused damage not only to flora and fauna, but also to human life (Cely, 2014; Lambert, 2017). In addition, the resulting long-running lawsuit received considerable media attention worldwide, this extended the environmental disaster; and its consequences are still present in the Ecuadorian population today (Krauss, 2014; Reuters, 2017). In contrast, young German people are virtually unaffected by such environmental disasters but are made aware of them and their consequences for people in other parts of the world almost daily by the media. Thus, we postulate that for Ecuadorian young people, the negative consequences of environmental problems for oneself are easier to imagine than for German young people. Due to these circumstances, the prioritization of egoistic motives for environmental concern in the Ecuadorian sample and altruistic motives in the German sample seems plausible.

While environmental concern has already been well researched across samples of different nationalities, there is a lack of cross-national empirical research regarding nature relatedness or equivalent constructs. Since nature relatedness is related to environmental concern, especially to biospheric concerns (Nisbet and Zelenski, 2013), the higher nature relatedness found in the Ecuadorian sample fits well with our result of the relative preference for biospheric over altruistic and egoistic environmental concerns in this sample. Nevertheless, the question arises as to how the different results come about in nature relatedness and the structure of the environmental concern. This question can be answered from two different directions. First, living in the biodiversity hotspot Tropical Andes may encourage Ecuadorian young people’s nature relatedness. Furthermore, the indigenous concept of *Buen Vivir*, which is not only deeply rooted in the culture of the indigenous people but also being politically instrumentalized (Lalander, 2016), may have an effect on the socialization process in Ecuador that could increase their nature relatedness. For example, the concept of *Buen Vivir* assumes a central position in the Constitution, in which the construction of “a new form of citizen coexistence in diversity and harmony with nature, to achieve good living (*Buen Vivir*)” (Asamblea Constituyente de Ecuador, 2008, p. 15) is announced. As a result, the indigenous guiding principles of *Buen Vivir* apply to all Ecuadorian citizens and not only to those of an indigenous background.

Second, the debate about *Buen Vivir* and the associated social awareness regarding environmental issues (Rieckmann et al., 2011; Lalander, 2016) may increase the pressure to respond in a socially desirable way (Schwartz et al., 1997; Smith, 2004). Both explanatory approaches probably apply to a certain extent. For instance, the items of the NR-6 “I always think about how my actions affect the environment” and “My connection to nature and the environment is a part of my spirituality” (Nisbet and Zelenski, 2013) are in many respects consistent with the concept of *Buen Vivir*, which is based on the idea of living in harmony with nature to achieve good living (*Buen Vivir)* and of interdependence of society and nature (Asamblea Constituyente de Ecuador, 2008; Vanhulst and Beling, 2014).

To summarize the results of the first research question, the current study showed that Ecuadorian students related more to nature than German students and were most concerned about the consequences of environmental problems for biospheric reasons, whereas German students were most concerned for altruistic reasons.

**Q2: How do young people’s gender and nationality, their basic human values, and time spent in nature affect their nature relatedness and environmental concern?**

**H2: Self-transcendence, time spent in nature, and having a female gender positively predict nature relatedness and biospheric environmental concern.**

Based on diverse results in the literature, in our second hypothesis, we assumed that self-transcendence (Sothmann and Menzel, 2017), time spent in nature (Mayer et al., 2009; Nisbet and Zelenski, 2013), and having a female gender (Stern et al., 1993; Tam, 2013) would predict nature relatedness. Although the regressions found that time spent in nature is a positive predictor for nature relatedness (Table 4), we must consider the ex post facto design of our study, which is why we cannot make a definitive statement about the direction of the relationship between the two variables. Indeed, it is also reasonable to assume that a sense of nature relatedness motivates people to seek out nature. Nonetheless, we hypothesized a positive effect of time spent in nature on nature relatedness on the basis of experimental studies that showed the positive effect of exposure to nature on college students’ nature connectedness (Mayer et al., 2009). However, it may be the case that there is a bidirectional relationship between these two variables, such as that having a desire to connect with nature leads to spending more time in nature, which in turn positively affects connectedness with nature and vice versa (see also Mayer et al., 2009; Nisbet et al., 2011; MacKerron and Mourato, 2013).

In accordance with available literature (Schultz, 2001), self-transcendence was the most powerful predictor for biospheric concern in both samples (Table 4). Among other things, self-transcendence represents a pro-environmental value orientation orientated toward the welfare of all living things and nature (universalism; Schwartz, 1992), which explains its positive effect on biospheric environmental concern and nature relatedness.

The positive effect of female gender on nature relatedness found in the German sample can be explained by Tam (2013), who found in an adult Chinese sample that female individuals had more dispositional empathy with nature, which was related to connection to nature. In contrast, in the Ecuadorian sample, female gender had a negative effect on nature relatedness, running contrary to our supposition and pointing to cultural differences regarding the relation between gender and nature relatedness.

The second part of our hypothesis dealt with biospheric environmental concern. As in the case of nature relatedness as dependent variable, self-transcendence and time spent in nature seemed to predict biospheric concern in both samples. However, the different sample sizes must be taken into account. It is very likely that time spent in nature in the German sample was significant only because of the very large sample size (*N* = 1,912). Such an effect would most likely not occur with a sample size comparable to the Ecuadorian sample. This also applies to the regression of time spent in nature on egoistic concern (Table 4).

Although it might seem surprising that female gender had a negative effect on biospheric environmental concern in the Ecuadorian sample, while there was no relation found in the German sample between these variables, Zelezny et al. (2000) came to comparable conclusions, examining gender differences in environmental attitudes and behaviors across 14 countries. They showed that only in three (Colombia, Ecuador, and El Salvador) out of the 14 countries did males have higher environmental attitudes than females. They also found that only in two of the 14 countries did males report higher ecocentric environmental attitudes than females (Dominican Republic and Ecuador). In addition to Ecuador, the mentioned study examined ten other Latin American countries, suggesting that Ecuador is an exception regarding gender differences in the human–nature relationship. Therefore, the findings of Zelezny et al. (2000) in an adult Ecuadorian sample could be replicated by our study for Ecuadorian young people, even if these differences cannot be explained easily.

Gender differences in environmental concern and nature relatedness can be explained by approaches based on gender roles and socialization, according to which behavior is a product of the socialization process, characterized by gender expectations in terms of cultural norms. Females are generally socialized to have a stronger “ethic of care” (Gilligan, 1982, p. 73), to be more compassionate, and to be more involved in caregiving activities than males (Beutel and Marini, 1995). Therefore, females are expected to be more empathic than males (Hoffman, 2008), which has been empirically proven (Baron-Cohen and Wheelwright, 2004; Jolliffe and Farrington, 2006). Based on these findings, Tam (2013) proposed that women have stronger dispositional empathy with nature than men do and could confirm his assumption in a study with Chinese adults. Based on this, gender differences in predicting nature relatedness and biospheric environmental concern could be an expression of culture-specific socialization, and it supports the hypothesis of Davidson and Freudenburg (1996) that gender differences in environmental concern are not universal.

As previously mentioned, we consider the indigenous concept of *Buen Vivir*, which is deeply rooted in the culture of the indigenous people, to be central in the explanation of nature relatedness and environmental concern. On a conceptual level, the variable of nature relatedness and the basic idea of *Buen Vivir* have many overlapping points and similarities (Nisbet et al., 2009). We propose that a life concept of living in harmony with nature that applies to everyone, male or female, influences the process of socialization. The current debate about *Buen Vivir* and the associated social awareness regarding environmental issues (Rieckmann et al., 2011; Lalander, 2016) may reinforce this effect. In addition, Rafael Correa, who was the President of Ecuador from 2007 to 2017 and promoted life in harmony with nature, may have been a role model for many Ecuadorian boys.

In summary, with regard to our second hypothesis we found that self-transcendence predicted students’ biospheric environmental concern in Germany and Ecuador. In addition, in the Ecuadorian sample, time spent in nature had a positive effect on biospheric concern, whereas female gender had a negative effect. No relation could be found in this respect in the German sample. In both samples, nature relatedness was predicted positively by self-transcendence and time spent in nature. Surprisingly, female gender predicted nature relatedness negatively in the Ecuadorian sample and positively in the German sample.

**H3: Self-transcendence positively predicts altruistic concern and negatively egoistic concern.**

With respect to our third hypothesis, self-transcendence was the only predictor for altruistic environmental concern, thus, our results are consistent with those in the literature (Schultz, 2001). As self-transcendence triggers prosocial norms oriented toward the welfare of humans (particularly through the value of benevolence) (Schwartz, 1992), its predictive power for altruistic environmental concern is plausible.

Surprisingly, we found self-transcendence to be a positive predictor for egoistic concern, even though Schultz (2001) and Schultz et al. (2005) found a negative relation between self-transcendence and egoistic environmental concern. However, the mentioned studies were conducted with adult samples, thus results are only applicable for adults. Sothmann and Menzel (2016) found that especially young people were shown to profit from nature as a resource for their own well-being and that this connection decreases with increasing age. Self-transcendence, especially the universalism value type, emphasizes the importance of caring for and adapting to nature, which represents the idea of the nature connection of including nature within the cognitive representation of self (Schultz, 2002). Accordingly, nature connected people are expected to relate the damage to their environment to themselves.

Therefore, it seems true that young people who are high in self-transcendence are concerned about environmental problems because of the biosphere and also because they are afraid of the destruction of the source for their own well-being and relate the damage to their environment to themselves.

However, we have to consider the low percentage of variance explained for egoistic concern by self-transcendence in Germany and Ecuador, which leads us to suspect that other variables besides self-transcendence and self-enhancement are more important in the explanation of egoistic environmental concern.

**H4: Self-enhancement positively predicts egoistic environmental concern.**

The results support our assumption that self-enhancement predicted egoistic environmental concern in both samples (Schultz, 2001; Schultz et al., 2005), because self-enhancement predicted egoistic environmental concern in both samples (Table 4). Self-enhancement reflects goals and ideals that are linked with tangible rewards for self (e.g., success, social power, enjoyment, and pleasure). We propose that people who are orientated toward self-enhancement values do not include other people or other living things within their representation of self (Schultz, 2001). Thus, our results replicated those of earlier studies conducted with adult samples from different countries (Schultz, 2001; Schultz et al., 2005).

## Conclusion

The aim of the present study was to compare Ecuadorian and German young people’s nature relatedness and environmental concern and to investigate its predicting factors. The following conclusions can be drawn from the results described in this article:

(1) Ecuadorian young people were found to be more related to nature than young people in Germany. Living in a biodiversity hotspot and culture-specific socialization are seen as reasons for the differences. However, a social desirability response bias cannot be ruled out, which is why we recommend the application of a scale to measure social desirability for further studies. (2) German and Ecuadorian young people differed in their structure of environmental concern. Living in a biodiversity hotspot, which includes the contact with biodiversity particularly worthy of protection, might be one explanation for the high biospheric environmental concern in the Ecuadorian sample. Differences between Ecuador and Germany regarding biodiversity loss and its immediately noticeable consequences served as an explanation for the high altruistic concern of German students and the high egoistic concern about the environmental problems of Ecuadorian students. (3) Gender differences between Ecuadorian and German young people in the explanation of nature relatedness and biospheric concern were found. These differences were interpreted as an expression of a culture-specific socialization. (4) Contrary to previous studies conducted with adult samples (Schultz et al., 2005), in our samples of young people, their self-transcendence had a positive effect on egoistic concern. We assume that young people will be better able than adults to combine the intrinsic value of nature with selfish goals, such using its positive effect on their well-being. (5) As in other studies conducted with adults, time spent in nature and self-transcendence also had positive effects for high school students’ nature relatedness and biospheric environmental concern.

Unlike a variety of previous studies conducted with adults, our results refer to the human–nature relationship of young people. The outcomes indicate that differences exist in the human–nature relationship between German young people, who live in an industrial country, and Ecuadorian young people, who live in a biodiversity hotspot. Nevertheless, the chosen variables could only explain a small proportion of the variance for the three dimensions of environmental concern, and thus our results should be validated with replication studies using a scale to measure social desirability. We assume that the students from Ecuadorian private schools are neither representative in terms of socio-ecological status, nor do they reflect cultural diversity of the country. Therefore, a sampling bias cannot be ruled out.

We assume that Ecuadorian students from private schools are more likely than those from public schools to have their basic material needs met. As the formation of environmental concern might be understood as a consequence of increasing post-materialism, private school students may differ from public school students in terms of their environmental concern (Maslow, 1954; Inglehart, 1995; Stern et al., 1999). On the basis of government expenditure per secondary school student for the year 2014, however, it can be seen that German students receive considerably more financial support from the state (11,180 US$) than do Ecuadorian secondary school students (338 US$; UNESCO Institute for Statistics [UIS], 2018; World Bank National Accounts data and OECD National Accounts data, 2018). For this reason, we assume that the comparison of German public school students with Ecuadorian private school students is more appropriate than with Ecuadorian public school students. Nevertheless, future studies should survey both private and public school students in order to assess for a possible sampling bias.

Nature relatedness and environmental concern, especially biospheric concern, are important prerequisites for pro-environmental behavior. In the face of a daily biodiversity loss, which is particularly prevalent in biodiversity hotspots, it is imperative to identify factors that contribute to the promotion of nature relatedness and biospheric environmental concern among young people. Our study clearly showed that young people living in Ecuador, a country that hosts two relevant biodiversity hotspots, were most concerned about the consequences of environmental problems for biospheric reasons. They also feel more related to nature than young people from an industrialized country such as Germany. In both samples self-transcendence was the strongest predictor for nature relatedness as well as for biospheric environmental concern. Hence it represents a particularly strong leverage point to stimulate pro-environmental behavior. Self-transcendence values could be fostered in both family life and teaching by addressing and rewarding aspects such as justice and solidarity instead of placing the focus on performance-oriented aspects.

The study indicated a clear positive effect of time spent in nature on biospheric concern only in the Ecuadorian sample. Living in a biodiversity hotspot and directly experiencing complex biotopes constitute a plausible reason for Ecuadorian young people’s high biospheric environmental concern and nature relatedness. As a consequence, also in countries with a relatively low biodiversity such as Germany, visiting and experiencing diverse biotopes, in or outside the country, could contribute to the promotion of both variables.

Finally, the effects of time spent in nature on nature relatedness emphasize the importance of giving young people opportunities to learn in and from nature, whether they are living in a biodiversity hotspot or an industrialized country. This can happen by means of family activities, leisure activities, or out-of-school environmental education. In the field of education, the results may encourage teachers to leave the classroom more often with their students and conduct environmental education directly in or close to nature in order to increase young people’s pro-environmental behavior.

## Data Availability

All data will be available in the Supplementary Material.

## Author Contributions

SM, J-NS, and MD did substantial contributions to the conception of the work. J-NS was responsible for the data acquisition in Germany and MD in Ecuador. MD interpreted the data and wrote the first draft of the work. SM and FF revised the work critically for important intellectual content and supported the statistical analyses.

## Conflict of Interest Statement

The authors declare that the research was conducted in the absence of any commercial or financial relationships that could be construed as a potential conflict of interest.
