# Supplementary material for: Nature Relatedness and Environmental Concern of Young People in Ecuador and Germany
Source: Front Psychol. 2019 Mar 7;10:453. doi: 10.3389/fpsyg.2019.00453 (PMC6416211; doi:10.3389/fpsyg.2019.00453)
Supplement: Supplementary file 1 [file Data_Sheet_1.zip › Supplementary Material/supplementary_material_nature_relatedness_and_environmental_concern_of_young_people_in_Ecuador_and_Germany.docx]

Supplementary Material

Nature relatedness and environmental concern of young people in Ecuador and Germany

Maximilian Dornhoff^1*^, Jan-Niklas Sothmann^1^, Florian Fiebelkorn^1^, Susanne Menzel^1^

*** Correspondence:** Maximilian Dornhoff: [dornhoff@biologie.uni-osnabrueck.de](mailto:dornhoff@biologie.uni-osnabrueck.de)

# Supplementary Tables

| **Supplementary Table 1 \|** Factor loadings based on a confirmatory factor analysis with MLR estimator for the Environmental Concern Scale (N_Germany_ = 2,064; N_Ecuador_ = 371). | | | | | | | |
| --- | --- | --- | --- | --- | --- | --- | --- |
| **Items** | **Germany** | | |  | **Ecuador** | | |
|  | **EGO** | **ALTRU** | **BIO** |  | **EGO** | **ALTRU** | **BIO** |
| ECEGO1: me | 0.58^InterA^ |  |  |  | 0.76 |  |  |
| ECEGO2: my lifestyle | 0.56^InterA^ |  |  |  | 0.69 |  |  |
| ECEGO3: my health | 0.73 |  |  |  | 0.71 |  |  |
| ECEGO4: my future | 0.71 |  |  |  | 0.58 |  |  |
| ECALTRU1: people in my community |  | 0.77^InterB^ |  |  |  | 0.81 |  |
| ECALTRU2: all people |  | 0.71 |  |  |  | 0.70 |  |
| ECALTRU3: children |  | 0.65 |  |  |  | 0.53^InterB^ |  |
| ECALTRU4: future generations |  | 0.73^InterB^ |  |  |  | 0.36^InterB^ |  |
| ECBIO1: plants |  |  | 0.81 |  |  |  | 0.85 |
| ECBIO2: marine life |  |  | 0.91 |  |  |  | 0.80 |
| ECBIO3: birds |  |  | 0.91 |  |  |  | 0.94 |
| ECBIO4: mammals |  |  | 0.85 |  |  |  | 0.81 |
| Robust CFI | 0.95 | | |  | 0.95 | | |
| Robust RMSEA | 0.08 | | |  | 0.08 | | |
| SRMR | 0.05 | | |  | 0.06 | | |
| *EC, environmental concern; EGO, egoistic; ALTRU, altruistic; BIO, biospheric;*  *Inter, added intercorrelation between items; CFI, Comparative Fit Index;*  *RMSEA, Root mean Square Error of Approximation;  SRMR, Standardized Root Mean Square Residual* | | | | | | | |

| **Supplementary Table 2 \|** Comparison between the uncentered and centered mean scores of the German and Ecuadorian samples. | | | | | | | | | | |
| --- | --- | --- | --- | --- | --- | --- | --- | --- | --- | --- |
| **Based on raw mean scores** | | | | | | | | | | |
| **Variables** | **Germany** | | |  | **Ecuador** | | | **t-test** | **95% BCaCI** | **Effect size *d*** |
|  | ***M*** | ***SE*** | ***SD*** |  | ***M*** | ***SE*** | ***SD*** |  |  |  |
| Egoistic EC | 3.87 | 0.02 | 0.74 |  | 4.40 | 0.03 | 0.65 | -15.07*** | [-0.61, -0.47] | 0.73 |
| Altruistic EC | 4.12 | 0.02 | 0.73 |  | 4.29 | 0.04 | 0.71 | -4.27*** | [-0.24, -0.09] | 0.23 |
| Biospheric EC | 4.01 | 0.02 | 0.91 |  | 4.50 | 0.03 | 0.72 | -12.32*** | [-0.58, -0.41] | 0.56 |
| ST | 3.83 | 0.01 | 0.55 |  | 4.09 | 0.03 | 0.62 | -8.15*** | [-0.33, -0.20] | 0.46 |
| SE | 3.00 | 0.02 | 0.69 |  | 3.05 | 0.04 | 0.74 | -1.24 | [-0.12, -0.03] | 0.07 |
| Nature relatedness | 2.66 | 0.02 | 0.78 |  | 3.69 | 0.04 | 0.83 | -24.54*** | [-1.12, -0.95] | 1.32 |
| Time spent in nature | 2.91 | 0.02 | 0.88 |  | 2.82 | 0.04 | 0.88 | 1.95* | [0,00, 1.18] | 0.10 |
| **Based on centered mean scores** | | | | | | | | | | |
| Egoistic EC | -0.14 | 0.02 | 0.74 |  | 0.02 | 0.03 | 0.65 | -3.53*** | [-0.20, -0.05] | 0.17 |
| Altruistic EC | 0.11 | 0.02 | 0.73 |  | -0.13 | 0.04 | 0.71 | 6.15*** | [0.17, 0.32] | 0.33 |
| Biospheric EC | 0.00 | 0.02 | 0.91 |  | 0.08 | 0.04 | 0.72 | -2.01* | [-1.61, -0.01] | 0.09 |
| ST | 0.39 | 0.01 | 0.55 |  | 0.47 | 0.03 | 0.62 | -2.55** | [-0.15, -0.02] | 0.14 |
| SE | -0.44 | 0.02 | 0.69 |  | -0.57 | 0.04 | 0.74 | 3.42** | [0.05, 0.21] | 0.19 |
| *EC, environmental concern; ST, self-transcendence; SE, self-enhancement; Confidence intervals based on 1000 bootstrap samples, *= p ≤ 0.05, **= p ≤ 0.01, ***= p ≤ 0.001.* | | | | | | | | | | |

| **Supplementary Table 3 \|** List of all used Items | |  |
| --- | --- | --- |
| **Item code** | **Item Formulation** | |
| **Time spent in nature** | | |
| TI1 | How much time do you spend in nature? | |
| **Environmental concern** | | |
| ECEGO1 | me | |
| ECEGO2 | my lifestyle | |
| ECEGO3 | my health | |
| ECEGO4 | my future | |
| ECALTRU5 | people in my community | |
| ECALTRU6 | all people | |
| ECALTRU7 | children | |
| ECALTRU8 | future generations | |
| ECBIO9 | plants | |
| ECBIO10 | marine life | |
| ECBIO11 | birds | |
| ECBIO12 | mammals | |
| **Portrait Value Questionnaire** | | |
| SEPO1 | It is important to him/her1 to be rich. He/She wants to have a lot of money and expensive things. | |
| SEPO2 | It is important to him/her to be in charge and tell others what to do. He/She wants people to do what he/she says. | |
| SEPO3 | He/She always wants to be the one who makes the decisions. He/She likes to be the leader | |
| SEAC1 | It is very important to him/her to show his/her abilities. He/She wants people to admire what he/she does. | |
| SEAC2 | Being very successful is important to him/her. He/She likes to impress other people. | |
| SEAC3 | Getting ahead in life is important to him/her. He/She strives to do better than others. | |
| SEHE1 | He/She seeks every chance he/she can to have fun. It is important to him/her to do things that give him/her pleasure. | |
| SEHE2 | Enjoying life’s pleasures is important to him/her. He/She likes to ‘spoil’ himself/herself. | |
| SEHE3 | He/She really wants to enjoy life. Having a good time is very important to him/her. | |
| STUN1 | He/She thinks it is important that every person in the world be treated equally. He/She believes everyone should have equal opportunities in life. | |
| STUN2 | It is important to him/her to listen to people who are different from him/her. Even when he/she disagrees with them, he/she still wants to understand them. | |
| STUN3 | He/She strongly believes that people should care for nature. Looking after the environment is important to him/her. | |
| STUN4 | It is important to him/her to adapt to nature and to fit into it. He/She believes that people should not change nature. | |
| STBE1 | It's very important to him/her to help the people around him/her. He/She wants to care for other people. | |
| STBE2 | It is important to him/her to be loyal to his friends. He/She wants to devote himself to people close to him. | |
| STBE3 | It is important to him/her to respond to the needs of others. He/She tries to support those he knows. | |
| STBE4 | Forgiving people who might have wronged him/her is important to him/her. He/She tries to see what is good in them and not to hold a grudge. | |
| *^1^In the German version, we used “the person” instead of “he/she” and “him/her”* | | |
| **NR-6** | | |
| NR1 | My connection to nature and the environment is a part of my spirituality. | |
| NR2 | My relationship to nature is an important part of who I am. | |
| NR3 | I feel very connected to all living things and the earth. | |
| NR4 | I always think about how my actions affect the environment | |
| NR5 | My ideal vacation spot would be a remote, wilderness area. | |
| NR6 | I take notice of wildlife wherever I am. | |
